# A Certain Sign for the Recognition of Cholera in the Early Stages

**Published:** 1873-08

**Authors:** 


### A certain sign for the recognition of Cholera in the early stages.

Dr. A. Hermann has a paper upon this subject in the June number of the *Allgem. Wien. Med. Zeitung*, which is translated in *The Clinic* of June 28 :

"All authors are agreed that the first urine after the collapse differs markedly from normal urine in *containing albumen, cylinders, hyaline and granular*, in abundance, and writers are tolerably unanimous in the opinion, that the danger of the typhoid stage is due to the kidney disease which is thus developed.

"The discovery of the author consists in the *detection of albumen, cylinders and epithelium in the urine during the time of the precursory diarrhœa.* If the examination of the urine show negative results, *cholera will not ensue.* The author has had now forty-eight cases in which to establish his discovery as a fact. In not one of these cases has he been deceived in his prognosis. It is very true that in *stadium asphycticum* little or no urine at all can be obtained for testing purposes ; yet if catheterization be regularly practised (and it should never be omitted in cholera) enough may be obtained at some time for chemical and microscopical examination. The author does not claim priority in the discovery of albumen in the urine of cholera collapse. Simon and

Hermann called attention to this fact in the third decade of the present century. The author's claim consists in the discovery of albumen in greater or less quantity during the stage of the '*diarrhœa premonitoria.*'

"Should an individual in health be suddenly attacked with diarrhœa, even during a cholera epidemic, *and should on examination no albumen be found in the urine, cholera will not develop*, not even if the individual be in such a position that he cannot protect himself in any way. Such a diarhœa may even be maltreated and still it will not lead to cholera, but will resolve itself in time, even though it may have reached such a grade as to have been called cholerine or even cholera.

"But if albumen be found in the urine of an individual suffering with diarhœa—with cylinders and kidney epithelium under the microscope—still the diagnosis of cholera is not absolute of course, as other affections may bring about this condition; yet as this manifestation is constant and never failing in cholera, as the collapse and typhoid condition are only observed in this condition, such a diarrhœa is to be diagnosticated as cholera when the other affections productive of albuminuria are excluded.

"It matters not whether the symptoms be light or severe, when albumen presents in the urine it establishes the existence of true cholera, and the graver symptoms will manifest in a short time.

"The author concludes his paper as follows: New observations have only confirmed me in the opinion before expressed that *however severe apparently choleraic the symptoms may be, the disease is not to be considered as true cholera if albumen be absent in the urine; on the other hand, the most anxious attention is to be directed to a case even in the earliest diarhœa when the urine is albuminous.*"
—*Phila. Med. Times.*

